# Relationship between the Regulation of Caspase-8-Mediated Apoptosis and Radioresistance in Human THP-1-Derived Macrophages

**DOI:** 10.3390/ijms19103154

**Published:** 2018-10-13

**Authors:** Hironori Yoshino, Haruka Konno, Koya Ogura, Yoshiaki Sato, Ikuo Kashiwakura

**Affiliations:** 1 Department of Radiation Science, Hirosaki University Graduate School of Health Sciences, 66–1 Hon-cho, Hirosaki, Aomori 036-8564, Japan; h18gg205@hirosaki-u.ac.jp (Y.S.); ikashi@hirosaki-u.ac.jp (I.K.); 2 Department of Radiological Technology, Hirosaki University School of Health Sciences, 66–1 Hon-cho, Hirosaki, Aomori 036-8564, Japan; haruknn@gmail.com (H.K.); k.ogura.h3@gmail.com (K.O.); 3 Graduate School of Education, Hirosaki University, 1 Bunkyo-cho, Hirosaki, Aomori 036-8560, Japan

**Keywords:** ionizing radiation, radioresistance, caspase-8, macrophages, apoptosis

## Abstract

Radiosensitivity varies depending on the cell type; highly differentiated cells typically exhibit greater radioresistance. We recently demonstrated that human macrophages derived from THP-1 monocytic cells, which lack *TP53*, are highly resistant to radiation-induced apoptosis compared with undifferentiated THP-1 cells. However, the mechanisms by which THP-1 cells acquire radioresistance during differentiation remain unknown. Herein, we investigated the mechanisms by which THP-1-derived macrophages develop p53-independent radioresistance by analyzing DNA damage responses and apoptotic pathways. Analysis of γ-H2AX foci, which indicates the formation of DNA double-strand breaks (DSB), suggested that a capacity to repair DSB of macrophages is comparable to that of radiosensitive THP-1 cells. Furthermore, treatment with inhibitors against DSB repair-related proteins failed to enhance radiation-induced apoptosis in THP-1-derrived macrophages. Analysis of the apoptotic pathways showed that radiosensitive THP-1 cells undergo apoptosis through the caspase-8/caspase-3 cascade after irradiation, whereas this was not observed in the macrophages. Caspase-8 protein expression was lower in macrophages than in THP-1 cells, whereas mRNA expressions were comparable between both cell types. Co-treatment with a proteasome inhibitor and ionizing radiation effectively induced apoptosis in macrophages in a caspase-8-dependent manner. Results suggest that the regulation of caspase-8-mediated apoptosis during differentiation plays a role in the p53-independent radioresistance of THP-1-derived macrophages.

## 1. Introduction

Radiation therapy is widely used for treatment of cancer. However, cytotoxicity of ionizing radiation against normal tissues limits the efficacy of radiation therapy. Ionizing radiation induces biological effects by causing DNA damage such as single- or double-strand breaks (DSB). Radiosensitivity varies depending on the cell type; for example, tissue stem/progenitor cells such as hematopoietic stem/progenitor cells are generally sensitive to ionizing radiation [1,2]. In contrast, non-proliferating and highly differentiated cells generally exhibit radioresistance. However, little is known about the mechanisms whereby highly differentiated cells become radioresistant.

Macrophages are innate immune cells found in the tissues, and they play key roles in the host’s defense against pathogens [3]. Bauer et al. reported that compared with monocytes, human monocyte-derived macrophages are more resistant to apoptosis caused by DNA damaging agents, including ionizing radiation [4,5]. This may be because macrophages express DNA repair proteins such as the DNA-dependent protein kinase, catalytic subunit (DNA-PKcs) during differentiation, whereas monocytes lack these proteins. Furthermore, they showed that the tumor suppressor gene p53, which plays critical roles in cellular DNA damage response pathways such as cell cycle arrest and apoptosis [6], mediates oxidative stress-induced apoptosis in monocytes [4]. These results suggest that macrophage radioresistance is a result of decreased p53-mediated apoptosis and increased capacity for DNA repair.

One of our recent studies demonstrated that macrophages derived from human THP-1 monocytic cells are highly resistant to radiation-induced apoptosis compared with undifferentiated THP-1 cells [7]. Since THP-1 cells lack the p53 gene [8], it is thought that the radioresistance of THP-1-derived macrophages is independent of the p53-mediated pathway. However, the mechanisms by which THP-1 cells acquire radioresistance during differentiation into macrophages remain unknown. We also demonstrated that both THP-1 cells and THP-1-derived macrophages undergo apoptosis after treatment with endoplasmic reticulum (ER) stress inducers [7], indicating that ER-mediated apoptotic pathways are functional in THP-1-derived macrophages. Other apoptotic pathways, such as mitochondria- and death receptor-mediated apoptotic pathways, are well studied [9,10] and are activated by ionizing radiation in many cell types [11,12]. Considering these results, we hypothesized that the radioresistance of THP-1-derived macrophages is due to the impairment of apoptotic pathways mediated by mitochondria and/or death receptors. In the present study, we investigated the p53-independent mechanisms of THP-1-derived macrophage radioresistance by focusing on DNA damage responses and apoptotic pathways. Herein, we show that ionizing radiation induces apoptosis in radiosensitive THP-1 cells through caspase-8-mediated pathways, whereas THP-1-derived macrophages are resistant to ionizing radiation rather due to impaired caspase-8-mediated apoptosis during differentiation and not to a capacity for DSB repair. These findings highlight the importance of caspase-8 in radiation-induced, p53-independent apoptosis, and will contribute to the relief of radiation-induced apoptosis in normal tissues as well as to the enhancement of apoptosis in radioresistant tumor cells.

## 2. Results

### 2.1. Effects of Ionizing Radiation on Apoptosis Induction in THP-1 Cells and THP-1-Derived Macrophages

**Figure 1:**
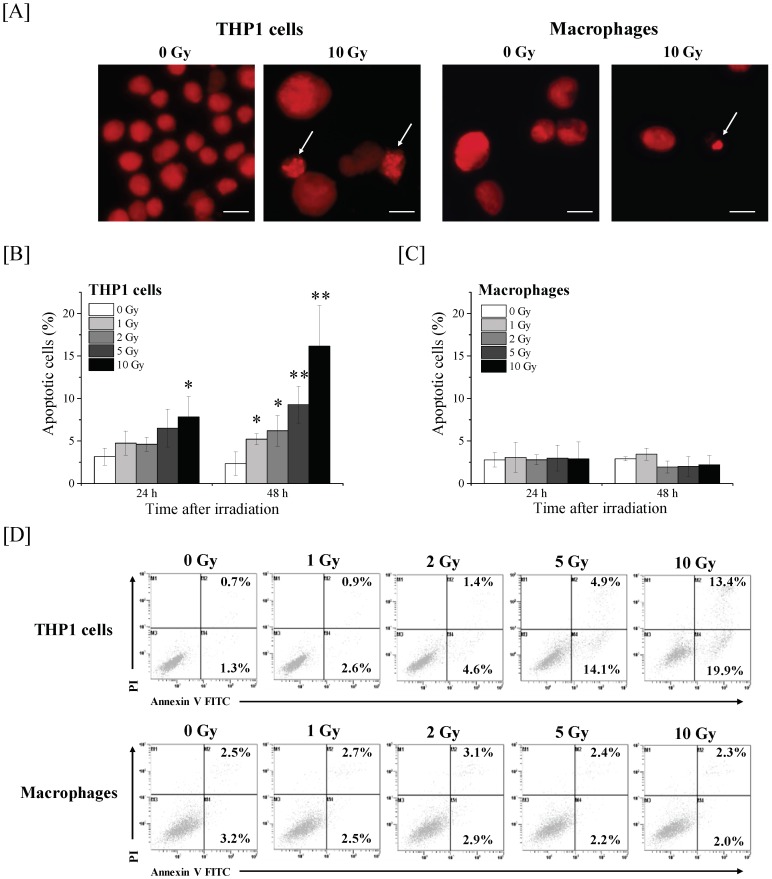
Effects of ionizing radiation on apoptosis induction in THP-1 cells and macrophages (**A**) THP-1 cells and macrophages irradiated with 10 Gy X-ray irradiation were cultured for 48 h and the nuclei morphology was analyzed to detect apoptosis. Representative nuclei morphologies are shown. Arrows indicate apoptotic cells. The bar in the figure is 20 µm in length. (**B**,**C**) THP-1 cells (**B**) and macrophages (**C**) were exposed to X-ray irradiation and then cultured for 24–48 h. After culture, the cells were harvested and evaluated for apoptosis. Data are presented as the mean ± SD of three independent experiments; * *p* < 0.05, ** *p* < 0.01. (**D**) THP-1 cells and macrophages were exposed to X-ray irradiation and then cultured for 48 h. After culture, the cells were harvested and evaluated for cell death by annexin V/PI staining. Representative cytograms are shown.

We first investigated the apoptosis induction of THP-1 cells and THP-1-derived macrophages (hereafter, macrophages) after irradiation. In 10 Gy-irradiated THP-1 cells, the percentage of apoptotic cells (identified by DNA fragmentation and chromatin condensation in their nuclei) was significantly higher than that in non-irradiated cells at 24 h after X-ray irradiation (Figure 1A). At 48 h post-irradiation, all tested doses (i.e., 1–10 Gy) significantly increased the number of apoptotic THP-1 cells, thus suggesting that ionizing radiation induces apoptosis in THP-1 cells in both time- and dose-dependent manners. However, in line with our previous report [7], no significant increase in apoptosis after irradiation was observed in the macrophages (Figure 1B). Similarly, analysis of cell death by annexin V/propidium iodide (PI) staining showed that ionizing radiation increased both annexin V+/PI– early apoptotic cells and annexin V+/PI+ late apoptotic/necrotic cells in THP-1 cells, whereas the increase in this population after X-ray irradiation was not observed in macrophages (Figure 1D). These results suggest that the macrophages acquired radioresistance during differentiation.

### 2.2. Kinetics of γ-H2AX in THP-1 and Macrophages after X-Ray Irradiation

**Figure 2:**
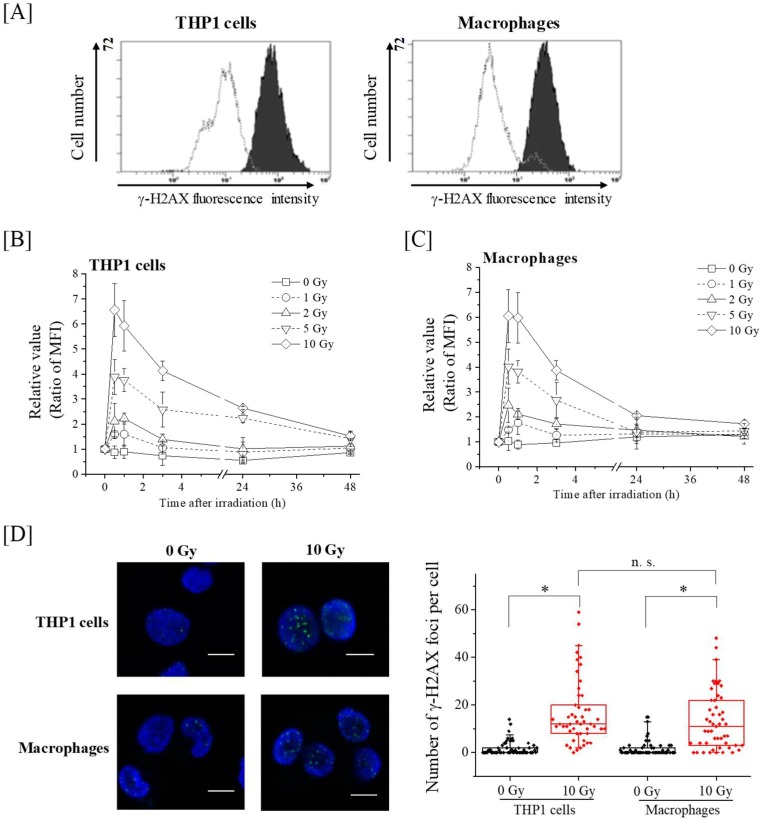
Kinetics of γ-H2AX expression in X-ray irradiated THP-1 cells and macrophages. (**A**) THP-1 cells and macrophages irradiated with 10-Gy X-ray irradiation were harvested 30 min after irradiation and the γ-H2AX expression was analyzed via flow cytometry. Representative histograms of γ-H2AX expression are shown. The dotted line histogram indicates the data from the non-irradiated cells, and the filled black histograms indicate the 10 Gy-irradiated cells. (**B**,**C**) THP-1 cells (**B**) and macrophages (**C**) were exposed to X-ray irradiation and cultured for 0.5–48 h. After culture, the cells were harvested and the γ-H2AX expression was analyzed via flow cytometry. The relative value of the γ-H2AX mean fluorescence intensity (MFI) from the irradiated cells compared with that of the pre-irradiation cells are shown. Data are presented as the mean ± SD of three independent experiments. (**D**) THP-1 cells and macrophages were exposed to 10-Gy X-ray irradiation and cultured for 24 h. After culture, the cells were harvested and the number of γ-H2AX foci was counted. (Left panel) Representative pictures of γ-H2AX foci are shown. Blue and green fluorescence indicate DAPI (nuclear stain) and γ-H2AX, respectively. The bar in the figure is 10 µm in length. (Right panel) Box charts of γ-H2AX foci number are shown. Bottoms and tops of the boxes are the 25th and 75th percentiles, respectively. The lines across the boxes are the median values. The ends of the whiskers represent 5th and 95th percentiles. The filled diamonds mean data of each cell. * and n.s. mean *p* < 0.01 and *p* > 0.05, respectively.

We next investigated the kinetics of the DSB marker γ-H2AX expression after X-ray irradiation. As shown in Figure 2, a dose-dependent increase in γ-H2AX expression after X-ray irradiation was observed in both radiosensitive THP-1 cells and radioresistant macrophages. In 1–10 Gy-irradiated THP-1 cells, the γ-H2AX expression peaked at 0.5–1 h after irradiation, and was approximately 1.5–6.5 fold higher than in non-irradiated cells. Similar to the results observed in the THP-1 cells, the γ-H2AX expression levels of 1–10 Gy-irradiated macrophages was approximately 1.5–6 fold higher than that of non-irradiated cells at 0.5–1 h after irradiation (Figure 2C). Although the γ-H2AX expression of the irradiated cells began to gradually decrease after 1 h, the γ-H2AX expression level of 10 Gy-irradiated THP-1 cells remained around 3-fold higher than that of non-irradiated control cells at 24 h after irradiation (Figure 2B). However, in macrophages, the increase in the γ-H2AX expression levels at 24 h after 10 Gy-irradiation was about 2-fold (Figure 2C). To clarify the difference in γ-H2AX between THP-1 cells and macrophages in detail, we counted the number of γ-H2AX foci at 24 h after 10 Gy-irradiation. As shown in Figure 2D, although the number of γ-H2AX foci in irradiated cells was significantly higher than that in non-irradiated cells, no significant difference in the number of γ-H2AX foci was observed between 10 Gy-irradiated THP-1 cells and macrophages. These results suggest that the radiation-induced DSB in the radioresistant macrophages are comparable to those of radiosensitive THP-1 cells.

### 2.3. Effects of DSB Repair-Related Proteins Inhibitors on the Apoptosis Induction in Macrophages

**Figure 3:**
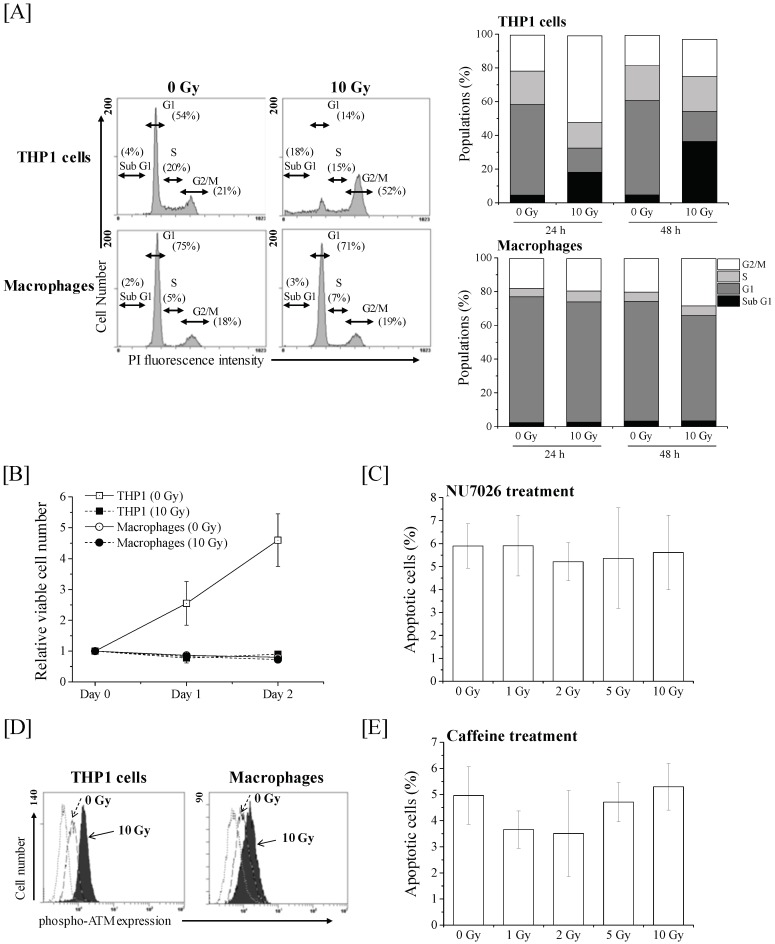
Effects of NU7026 and caffeine on apoptosis induction in macrophages. (**A**) THP-1 cells and macrophages irradiated with 10-Gy X-ray irradiation were harvested 24–48 h after irradiation for cell cycle analysis. (Left panel) Representative cell cycle histograms at 24 h after irradiation are shown. (Right panel) Populations of each cell cycle phase at 24–48 h after 10-Gy X-ray irradiation are shown. (**B**) THP-1 cells and macrophages irradiated with 10-Gy X-ray irradiation were harvested 24–48 h after irradiation, and viable cell number was counted by trypan blue dye exclusion assays. Data are presented as the mean ± SD of three independent experiments. (**C**) Macrophages pretreated with NU7026 were exposed to 10-Gy X-ray irradiation. The cells were cultured for 48 h and harvested for the detection of apoptosis. Data are presented as the mean ± SD of three independent experiments. (**D**) THP-1 cells and macrophages irradiated with 10-Gy X-ray irradiation were cultured for 0.5 h and harvested for analysis of intracellular phosphorylated-ATM expression. Representative histograms are shown. The dotted line histogram indicates cells stained with isotype control. The broken line and filled gray histograms indicate the phosphorylated-ATM expression in non-irradiated cells and 10-Gy X-ray-irradiated cells, respectively. (**E**) Macrophages pretreated with caffeine were exposed to 10-Gy X-ray irradiation. The cells were cultured for 72 h and harvested for the detection of apoptosis. Data are presented as the mean ± SD of three independent experiments.

Since ionizing radiation induces biological effects by causing DNA damage such as DSB, we next investigated the involvement of DSB repair-related proteins in the radioresistance of macrophages. DSB are repaired by two major pathways as follows: homologous recombination (HR) and non-homologous end joining (NHEJ) [13]. HR repair depends on the cell cycle phase, functioning only during the S and G_2_ phases, whereas the NHEJ repair functions are irrespective of the cell cycle phase [14]. Therefore, we analyzed the cell cycle profile of THP-1 cells and macrophages after 10 Gy X-ray irradiation. As shown in Figure 3A, the 10 Gy-irradiated THP-1 cells were primarily in the G_2_/M phase at 24 h after irradiation, and followed by increase in sub-G1 population, which contains cells with fragmented DNA and is a hallmark of apoptosis, at 48 h after irradiation. In terms of macrophages, they were in the G1 phase and the proportion of S phase was lower compared with THP-1 cells, which may be related to the non-proliferating property of macrophages (Figure 3B). Similar to the cell cycle profile of non-irradiated macrophages, the 10 Gy-irradiated macrophages were also in the G1 phase (Figure 3A). Taken together, these results suggest that the DSB repair of macrophages takes place primarily via the NHEJ pathway. Therefore, we investigated the involvement of NHEJ on the radioresistance of macrophages using NU7026, an inhibitor of DNA-PKcs, which plays a crucial role in NHEJ repair. No significant increase in radiation-induced apoptosis was observed in the macrophages treated with NU7026 (Figure 3C). Since the phosphorylated-ataxia–telangiectasia mutated (ATM) expression, which plays a key role in DNA damage response and is involved in HR repair [15], was observed in both the 10 Gy-irradiated THP-1 cells and macrophages (Figure 3D), we also investigated the involvement of ATM on the radioresistance of macrophages using an ATM/ataxia–telangiectasia and Rad-3-related (ATR) inhibitor caffeine [16]. As a result, no significant increase in radiation-induced apoptosis was observed in the macrophages treated with caffeine (Figure 3D). Therefore, it is unlikely that the radioresistance of THP-1-derived macrophages is due to their DSB repair capacity via NHEJ and HR repair.

### 2.4. Ionizing Radiation Induces Apoptosis in THP-1 Cells through the Caspase-8/Caspase-3 Pathway

**Figure 4:**
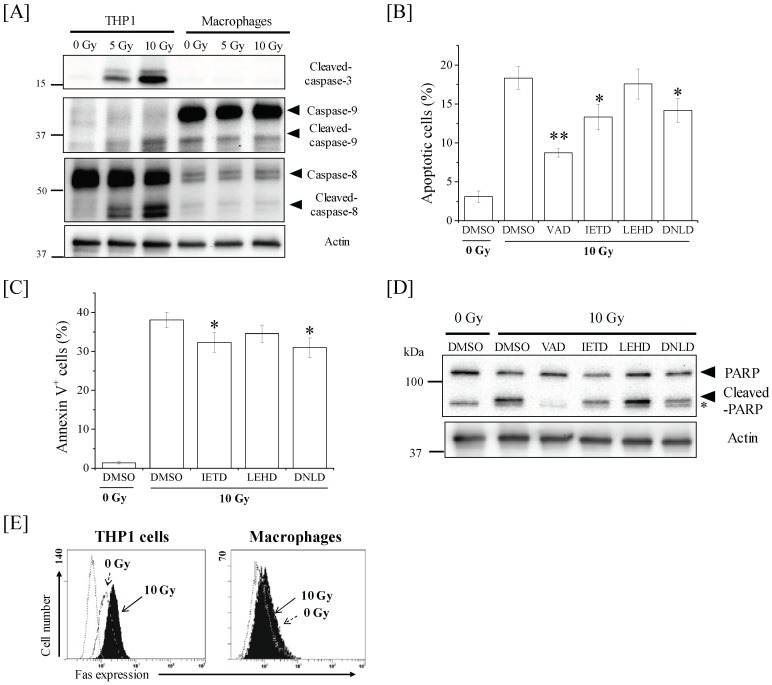
Involvement of caspases in X-ray-irradiated THP-1 cells. (**A**) THP-1 cells and macrophages were exposed to 10-Gy X-ray irradiation. The cells were cultured for 48 h and then harvested for Western blot analyses of caspase-3, -8, and -9. The expression of β-actin was analyzed as a loading control. (**B**–**D**) THP-1 cells pretreated with caspase inhibitors or dimethyl sulfoxide (DMSO) were exposed to 10-Gy X-ray irradiation. The cells were cultured for 48 h and harvested for the detection of apoptosis (**B**), cell death analyses (**C**), and Western blot analyses (**D**). (**B**,**C**) Data are presented as the mean ± SD of three independent experiments. * *p* < 0.05, ** *p* < 0.01; comparisons made against DMSO +10-Gy irradiation. (**D**) Representative data from the Western blotting of poly ADP ribose polymerase (PARP) are shown. The expression of β-actin was analyzed as a loading control. The asterisk indicates a non-specific band. (**E**) THP-1 cells and macrophages exposed to 10-Gy X-ray irradiation were cultured for 24 h and 48 h, respectively. The cells were harvested for the analysis of cell surface Fas expression. Representative histograms are shown. The dotted line histogram indicates cells stained with isotype control. The broken line and filled gray histograms indicate the Fas expression in non-irradiated cells and 10-Gy X-ray-irradiated cells, respectively.

To elucidate the mechanism resulting in the radioresistance of THP-1-derived macrophages, we next investigated the caspase-dependent apoptotic pathways. Cysteine protease caspase plays key roles in apoptosis induction, and caspase-8 and caspase-9 are involved in apoptosis mediated by death receptors and mitochondria, respectively [17]. Therefore, we first analyzed the active form of caspase-3, -8, and -9. As shown in Figure 4A, X-ray irradiation dramatically increased the expression of the cleaved active forms of caspase-3 and caspase-8 in THP-1 cells, whereas the increase in cleaved caspase-9 expression was slight. The radiation-induced apoptosis of THP-1 cells was significantly suppressed when THP-1 cells were treated with Z-Val-Ala-Asp (OMe)-CH_2_F (Z-VAD-fmk; a pan-caspase inhibitor), Ac-Asp-Asn-Leu-Asp-H (aldehyde) (Ac-DNLD-cho; a caspase-3 inhibitor), or acetyl-Ile-Glu-His-Asp-aldehyde (Ac-IETD-cho; a caspase-8 inhibitor), but not when they were treated with acetyl-Leu-Glu-His-Asp-H-aldehyde (Ac-LEHD-cho; a caspase-9 inhibitor) (Figure 4B). In line with the results of apoptosis, treatment with Ac-IETD-cho and Ac-DNLD-cho, not Ac-LEHD-cho, significantly suppressed the radiation-induced annexin V+ dead cells of THP-1 cells (Figure 4C). Furthermore, treatment with Ac-IETD-cho, Ac-DNLD-cho, and Z-VAD-fmk decreased the 10-Gy irradiation-induced expression of cleaved poly ADP ribose polymerase (PARP), which is a substrate for cleaved caspase-3 (Figure 4D). These results suggest that ionizing radiation induces the apoptosis of THP-1 cells through the caspase-8/caspase-3 pathway. Since death receptors such as Fas can activate the caspase-8-mediated apoptotic pathway, we also investigated the expression of Fas in X-ray-irradiated THP-1 cells. As shown in Figure 4E, 10-Gy X-ray irradiation dramatically increased the cell surface Fas expression, thus suggesting the potential involvement of Fas in the activation of caspase-8 in X-ray-irradiated THP-1 cells. However, in contrast to the results observed in THP-1 cells, no clear increase in the expression of cleaved caspase-3, -8, -9, or cell surface Fas was observed in X-ray-irradiated macrophages (Figure 4A). Interestingly, the expression levels of both procaspase-8 and cleaved caspase-8 in THP-1-derived macrophages were clearly lower than those in THP-1 cells.

### 2.5. Relationship between the Radioresistance of Macrophages and Caspase-8

We next investigated the relationship between the radioresistance of macrophages and caspase-8 expression.

**Figure 5:**
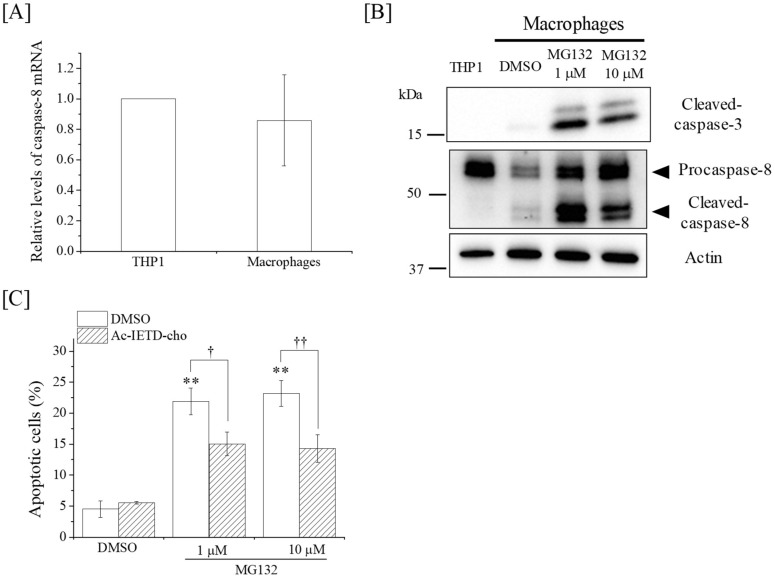
Effects of MG132 on the caspase-8 expression and apoptosis induction in macrophages. (**A**) The caspase-8 mRNA expression of THP-1 cells and macrophages were analyzed using quantitative reverse transcription polymerase chain reaction (qRT-PCR). Data are presented as the mean ± SD of four independent experiments. (**B**) Macrophages were cultured in the presence of the proteasome inhibitor MG132 or DMSO. The cells were cultured for 24 h and harvested for Western blot analyses of caspase-3 and -8. The expression of β-actin was analyzed as a loading control. (**C**) Macrophages pretreated with the caspase-8 inhibitor Ac-IETD-cho or DMSO were cultured in the presence of MG132. The cells were cultured for 24 h and harvested for the detection of apoptosis. Data are presented as the mean ± SD of three independent experiments. ** *p* < 0.01 vs. DMSO control. † and †† indicate *p* <0.05 and *p* <0.01, respectively.

First, the mRNA expression of caspase-8 was investigated. As shown in Figure 5A, the expression of caspase-8 mRNA in macrophages is comparable to that in THP-1 cells, suggesting that the observed downregulation of caspase-8 protein expression is not due to the downregulation of caspase-8 mRNA. Therefore, we next investigated the effects of the proteasome inhibitor MG132 on the expression levels of caspase-8. As shown in Figure 5B, treatment with MG132 increased the expression levels of both procaspase-8 and cleaved caspase-8. Interestingly, treatment with MG132 induced apoptosis in macrophages, and this apoptosis induction was significantly suppressed by Ac-IETD-cho (Figure 5C). These results suggest that inhibition of caspase-8 degradation induces apoptosis in macrophages in a caspase-8-dependent manner.

**Figure 6:**
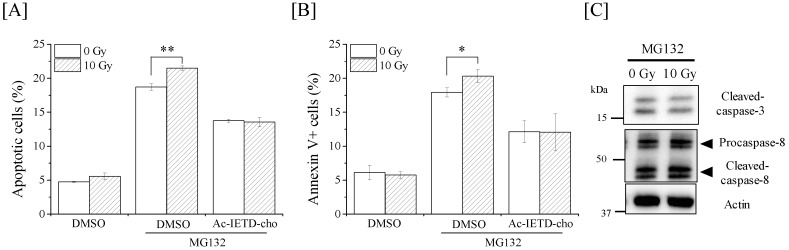
Effects of co-treatment with MG132 and ionizing radiation on apoptosis induction in macrophages. (**A**,**B**) Ac-IETD-cho or DMSO were added to the culture medium 1 h before the addition of MG132. One hour after the addition of MG132 (1 µM), the cells were exposed to 10-Gy X-ray irradiation. The cells were cultured for 24 h and harvested for the detection of apoptosis and cell death analyses. Data are presented as the mean ± SD of three independent experiments. * *p* < 0.05, ** *p* < 0.01. (**C**) MG132 (1 µM) were added to the culture medium 1 h before 10-Gy X-ray irradiation. The cells were cultured for 24 h and harvested for Western blot analyses of caspase-3 and -8. The expression of β-actin was analyzed as a loading control.

Finally, we examined whether co-treatment with MG132 and X-ray irradiation enhances apoptosis in macrophages. Co-treatment with MG132 and 10-Gy X-ray irradiation enhanced apoptosis in macrophages, and the increase in apoptotic cells was inhibited by the caspase-8 inhibitor Ac-IETD-cho (Figure 6A). Similar results were confirmed in analyses of annexin V+ dead cells (Figure 6B). Taken together, these results suggest the relationship between the radioresistance of THP-1-derived macrophages and caspase-8. However, the expression of active caspase-3 and -8 in the cells co-treated with MG132 and 10-Gy X-ray irradiation was comparable to that in the cells treated with MG132 alone (Figure 6C).

## 3. Discussion

In radiation biology, it is understood that non-proliferating and highly differentiated cells exhibit radioresistance, but little is known about the mechanisms by which these cells acquire radioresistance during differentiation. In the present study, we investigated the p53-independent radioresistance mechanisms of THP-1-derived macrophages. We demonstrated that ionizing radiation induces apoptosis in radiosensitive THP-1 cells through the caspase-8/caspase-3 pathway, and that this apoptosis pathway is not activated in X-ray-irradiated radioresistant macrophages. We also found that the caspase-8 protein expression decreased during macrophage differentiation. Furthermore, co-treatment with the proteasome inhibitor MG132 and X-ray irradiation enhanced apoptosis in the macrophages, and the increase in apoptotic cells was inhibited by caspase-8 inhibitors, thus suggesting the relationship between the radioresistance of THP-1-derived macrophages and caspase-8. It was reported that caspase-8 expression plays a role in apoptosis resistance induced by tumor necrosis factor-related apoptosis-inducing ligand, chemotherapeutic agents, and ionizing radiation [18,19,20,21]. Tsurushima et al. reported that overexpression of caspase-8 effectively enhanced radiation-induced cytotoxic effects, including apoptosis [21]. In addition, Afshar et al. showed that inhibition of caspase-8 expression by siRNA decreased the radiation-induced apoptosis in glioma cells [20]. Therefore, it is possible that the downregulation of caspase-8 expression during differentiation of THP-1 cells leads to the radioresistance of THP-1-derived macrophages.

Since nuclear DNA is the primary target of ionizing radiation, responses to and repair of this DNA damage may affect the cellular outcomes from ionizing radiation. The cells with DNA damage undergo cell cycle arrest to repair DNA damage, or apoptosis if DNA damage is too severe. In the present study, non-proliferating macrophages were primarily in G1 phase with or without X-ray irradiation, while THP-1 cells with proliferation ability underwent G2/M arrest after X-ray irradiation, which was followed by apoptosis. Some reports indicate that DNA damaging agents including ionizing radiation induce apoptosis following G2/M arrest [22,23,24]. Therefore, it is likely that G2/M arrest is one of the key events determining cell fate after DNA damage, and that attenuation of G2/M arrest after differentiation contributes to the radioresistance of non-proliferating macrophages.

DSB are severe DNA damage induced by ionizing radiation, and DSB repair is closely related to cell survival after radiation exposure. For example, it was reported that inhibition of DSB repair-related proteins such as DNA-PKcs and ATM enhances radiosensitivity [16,25,26,27]. In addition, Bauer et al. reported that human macrophages express DNA repair proteins, including DNA-PKcs, during differentiation, which contributes to their resistance to DSB induced by DNA damaging agents, including ionizing radiation [5]. It was reported that THP-1-derived macrophages also express DNA-PKcs and other DNA repair proteins during differentiation [28], as do macrophages differentiated from human primary monocytes. Therefore, we hypothesized that the high DNA repair capacity of THP-1-derived macrophages plays a role in the radioresistance of those cells. However, no significant difference in the number of γ-H2AX foci was observed between 10 Gy-irradiated THP-1 cells and macrophages. In addition, the DNA-PKcs inhibitor NU7026 and ATM/ATR inhibitor caffeine did not greatly affect radiation-induced apoptosis in macrophages. Therefore, although we need to investigate the difference in the expression and activation of DNA repair-related proteins such as DNA-PK and ATR between THP-1 cells and macrophages in detail, it is thought that the relationship between the radioresistance of THP-1-derived macrophages and DNA damage response is low. THP-1 cells lack *TP53*, a tumor suppressor gene that plays crucial roles in DNA damage responses, including apoptosis induction. Therefore, the failure of NU7026 or caffeine to enhance radiation-induced apoptosis in THP-1-derived macrophages is due to the loss of the p53-mediated DNA damage response.

Although it is understood that the p53 network is profoundly involved in apoptosis induction through the actions of various stimuli including ionizing radiation [29], we found that ionizing radiation induces apoptosis in THP-1 cells through caspase-8/caspase-3 activation in a p53-independent manner. Yu et al. reported that ionizing radiation induces the activation of caspase-3 and apoptosis in human lymphoblast cell lines through both p53-dependent and p53-independent pathways [30]. Furthermore, Afshar et al. reported results similar to those of the present study—that ionizing radiation induces caspase-8-mediated apoptosis in glioma cells in a p53-independent manner [20]. The death receptor-mediated apoptotic pathway is known to induce caspase-8-mediated apoptosis, and several studies have shown that DNA damaging agents, including ionizing radiation, increase the expression of death receptors, such as death receptor 5 and Fas, through both p53-dependent and p53-independent pathways [31,32]. We observed an upregulation of Fas expression in THP-1 cells, not macrophages, after irradiation. Therefore, it is likely that ionizing radiation activates caspase-8 through upregulation of death-receptor expression in THP-1 cells, and that the loss of Fas upregulation in X-ray-irradiated macrophages may contribute to the radioresistance of macrophages. The involvement of Fas in radiation-induced apoptosis in THP-1 cells must be clarified in a future study.

Kiener et al. reported that human macrophages derived from primary monocytes show an increase in resistance to Fas-induced apoptosis upon differentiation, and indicated that a site downstream of the Fas receptor–ligand interaction contributes to the difference in sensitivity to Fas-induced apoptosis between monocytes and macrophages [33]. In the present study, we showed that the expression of caspase-8 protein in macrophages was lower than that in THP-1 cells, while no significant difference in the caspase-8 mRNA expression between THP-1 cells and macrophages was observed. We also found that treatment with the proteasome inhibitor MG132 induced apoptosis in radioresistant macrophages through caspase-8 activation and subsequent increases in caspase-8 protein expression. Similar to our results, other reports have shown that proteasome inhibitors, including MG132, induce caspase-8-mediated apoptosis in various cancer cell lines [34,35]. In addition, it was reported that caspase-8 stabilization after proteasome inhibition is observed in some cancer cells [36,37]. Therefore, it is possible that the stabilization of caspase-8 protein expression is important for the induction of apoptosis by proteasome inhibitors and/or ionizing radiation, and that the loss of stabilization of caspase-8 protein expression during differentiation contributes to the radioresistance of THP-1-derived macrophages. Since tumor necrosis factor receptor-associated factor 2 (TRAF2) is thought to play a role in the proteasomal degradation of caspase-8 by promoting K48-linked ubiquitination [38], the role of TRAF2 in the downregulation of caspase-8 protein expression during differentiation of THP-1 cells needs to be investigated in a future study.

In the present study, although caspase-8 inhibitor inhibited the increase in apoptotic cells and annexin V+ cells in macrophages by co-treatment with MG132 and X-ray irradiation, no clear increase in the cleaved caspase-3 and -8 expressions by co-treatment was observed. It is known that apoptosis is tightly regulated by not only pro-apoptotic molecules but also anti-apoptotic molecules. The inhibitor of the apoptosis proteins (IAPs) family is a potent inhibitor of caspases activities, and can regulate cell death including apoptosis [39]. For example, X-linked inhibitor of apoptosis protein (XIAP), which is one of the IAPs family, can directly inhibit the activity of processed forms of caspase-3 [39]. Yang et al. reported that DNA damage can induce the depletion of IAPs including XIAP [40]. Therefore, in the condition that caspase-8 expression was restored and activated by treatment with MG132, ionizing radiation might enhance caspase-8-mediated apoptosis in macrophages by modulating IAPs expression. The expression of IAPs in macrophages needs to be investigated in a future study.

In summary, the present study indicates that ionizing radiation induces apoptosis in THP-1 cells through p53-independent, caspase-8/caspase-3 apoptotic pathways, whereas THP-1-derived macrophages show resistance to ionizing radiation because caspase-8-mediated apoptosis is impaired during differentiation. These findings highlight the importance of capsase-8 in radiation-induced, p53-independent apoptosis, and will contribute to the relief of radiation-induced apoptosis in normal tissues, as well as to the enhancement of apoptosis in radioresistant tumor cells.

## 4. Materials and Methods

### 4.1. Reagents

Phorbol 12-myristate 13-acetate (PMA), PI, caffeine, and DMSO were purchased from Sigma-Aldrich (St. Louis, MO, USA). Z-VAD-fmk, Ac-LEHD-cho, Ac-IETD-cho, and Ac-DNLD-cho were purchased from Peptide Institute, Inc. (Osaka, Japan). Cleaved caspase-3 antibody (#9661), caspase-8 antibody (#9746), caspase-9 antibody (#9502), PARP (46D11) rabbit monoclonal antibody (#9532), β-actin antibody (#4967), anti-rabbit IgG horseradish peroxidase (HRP)-linked antibody (#7074), anti-mouse IgG HRP-linked antibody (#7076), phosphor-ATM (Ser1981) (D25E5) antibody (#13050), and Alexa Fluor^®^ 488-conjugated goat anti-rabbit IgG (#4412) were purchased from Cell Signaling Technology Japan, K.K. (Tokyo, Japan). The fluorescein isothiocyanate (FITC)-labeled monoclonal antibody anti-human cluster of differentiation 95 (CD95-FITC) was purchased from BioLegend (San Diego, CA, USA). FITC-conjugated mouse IgG_1_ antibody was purchased from Beckman–Coulter (Fullerton, CA, USA).

### 4.2. Cell Culture and Treatment

THP-1 human acute monocytic leukemia cells were obtained from RIKEN Bio-Resource Center (Tsukuba, Japan). The cells were cultured in RPMI1640 supplemented with 1% penicillin and streptomycin (Gibco, Grand Island, NY, USA) and 10% heat-inactivated fetal bovine serum (Japan Bioserum Co., Ltd., Fukuyama, Japan) at 37 °C in a humidified atmosphere containing 5% CO_2_. THP-1-derived macrophages were prepared as previously described [41]. THP-1 cells (2.0 × 10^5^ cells/mL) were plated in 35-mm dishes (Iwaki, Tokyo, Japan) with 2 mL of medium containing 100 ng/mL PMA and cultured for 48 h. After 48 h, the medium containing PMA was replaced with PMA-free fresh medium and the macrophages were then used in subsequent experiments.

### 4.3. In Vitro X-Ray Irradiation

X-ray irradiation (150 kVp, 20 mA, 0.5 mm Al, and 0.3-mm Cu filters) was performed using an X-ray generator (MBR-1520R-3; Hitachi Medical Corporation, Tokyo, Japan) at a distance of 45 cm from the focus, with a dose rate of 1.00–1.04 Gy/min. In some experiments, the pan-caspase inhibitor Z-VAD-fmk (50 µM), the caspase-8 inhibitor Ac-IETD-cho (100 µM), the caspase-9 inhibitor Ac-LEHD-cho (100 µM), the caspase-3 inhibitor Ac-DNLD-cho (100 µM), the DNA-PKCs inhibitor NU7026 (5 µM) or the ATM/ATR inhibitor caffeine (2 mM) was added 1 h before X-ray irradiation.

### 4.4. Detection of Apoptotic Cells

The harvested cells were fixed with 1% glutaraldehyde/PBS solution overnight at 4 °C. The fixed cells were washed with PBS(−), pH 7.4, and resuspended in PBS(−) containing 40 μg/mL PI for 15 min in the dark at room temperature. At least 200 cells were scored with reflected-light fluorescence using an Olympus IX51 microscope (Olympus Optical Co., Ltd., Tokyo, Japan); cells with chromatin condensation and fragmentation were scored as apoptotic cells.

### 4.5. Cell Death Analysis

Cell death was analyzed by annexin V-FITC (BioLegend Inc., San Diego, CA, USA) and PI staining according to the manufacturer’s instructions. In brief, the cells treated with each compound were harvested, washed, and suspended in annexin V Binding Buffer (BioLegend). The annexin V-FITC (2.5 µg/mL) and PI solution (50 µg/mL) were added to the cell suspension and incubated for 15 min at room temperature in the dark. Then, the apoptotic cells were analyzed via flow cytometry (Cytomics FC500; Beckman–Coulter, Inc., Brea, CA, USA).

### 4.6. Detection of γ-H2AX by Flow Cytometry

Gamma-H2AX expression was analyzed via flow cytometry as previously described [42]. In brief, the harvested cells were fixed with ice-cold 70% methanol overnight at −20 °C. The fixed cells were washed with wash buffer (WB; PBS, pH 7.4, containing 0.5% bovine serum albumin) and treated with WB containing 0.25% Triton-X 100 on ice for 5 min. After washing with WB, the cell pellets were incubated with an anti-phospho-histone H2AX monoclonal antibody (JBW301; Upstate Biotechnology, Lake Placid, NY, USA) at a 300-fold dilution in WB containing 0.25% Triton-X 100 at room temperature for 1 h. The labeled cells were washed with WB and treated with an AlexaFluor 488^®^-conjugated anti-mouse IgG secondary antibody (Molecular Probes, Eugene, OR, USA) at a 400-fold dilution in WB containing 0.25% Triton-X 100 for 1 h in the dark. The stained cells were washed with WB and analyzed via flow cytometry (Cytomics FC500; Beckman Coulter, Inc., Brea, CA, USA).

### 4.7. γ-H2AX Foci Analysis

The harvested cells were fixed with ice-cold 70% ethanol overnight at −20 °C. The fixed cells were washed with PBS(−), permeabilized in 0.5% Triton X-100 on ice for 5 min, and washed twice with PBS(−). The cells were then incubated with an anti-phospho-histone H2AX monoclonal antibody diluted 1:300-fold with 20 mM Tris-HCl (pH 7.4), 137 mM NaCl, 0.1% TWEEN-20 (TBST) containing 5% skimmed milk at 37 °C for 120 min, and subsequently washed with PBS(−) and incubated with AlexaFluor 488^®^-conjugated anti-mouse IgG secondary antibody (#4408, Cell Signaling Technology Japan, K.K.) diluted 1:400-fold with TBST containing 5% skimmed milk at 37 °C for 60 min. Following a second wash with PBS(−), the cells were adhered to microscope glass slides using a StatSpin^®^ CytoFuge 2 (Iris Sample Processing, Inc., Westwood, MA, USA) and mounted using Vectashield^®^ Mounting Medium with DAPI (Vector Laboratories, Inc., Burlingame, CA, USA). For the quantitative analysis, the γ-H2AX foci were counted per cell using an LSM 710 laser scanning microscope (Carl Zeiss Microscopy Co., Ltd., Tokyo, Japan). The number of γ-H2AX foci per cell was counted for more than 50 cells in every sample.

### 4.8. Cell Cycle Analysis

Cell cycle analysis was performed as previously reported [43]. The cell cycle distribution was analyzed using flow cytometry (Cytomics FC500; Beckman Coulter).

### 4.9. Intracellular Phosphorylated-ATM Staining

The harvested cells were washed once with PBS(−) and were fixed in 4% formaldehyde (Sigma–Aldrich, Merck KGaA, Darmstadt, Germany) for 10 min at 37 °C. After washing with PBS(−), the cells were permeabilized with 90% methanol overnight at −20 °C. After washing with incubation buffer (PBS containing 0.5% bovine serum albumin), cells were incubated with a primary phosphorylated-ATM antibody diluted 1:1600-fold with incubation buffer for 1 h at room temperature. Following a second wash with incubation buffer, cells were stained with Alexa Fluor^®^ 488-conjugated anti-rabbit secondary antibody diluted 1:400-fold with incubation buffer for 30 min at room temperature in the dark. As a control, cells were stained with Alexa Fluor^®^ 488-conjugated secondary antibody alone. After 30 min, cells were washed with incubation buffer and were analyzed via flow cytometry (Cytomics FC500; Beckman Coulter, Inc., Brea, CA, USA).

### 4.10. SDS-PAGE and Western Blotting

SDS-PAGE and Western blotting were performed as previously reported [7]. The following primary antibodies were used: anti-cleaved caspase-3 rabbit antibody (1:3000), anti-caspase-8 mouse antibody (1:3000), anti-caspase-9 rabbit antibody (1:3000), anti-PARP rabbit antibody (1:3000), or anti-β-actin rabbit antibody (1:4000). The following secondary antibodies were used: HRP-linked anti-rabbit IgG antibody (1:10,000) or HRP-linked anti-mouse IgG antibody (1:10,000). The antigens were visualized using Clarity^TM^ Western ECL Substrate (Bio-Rad Laboratories, Inc., Hercules, CA, USA). Blot stripping was performed using Stripping Solution (Wako Pure Chemical Industries, Ltd., Osaka, Japan).

### 4.11. Analysis of Cell Surface Fas Expression

The analysis of cell surface Fas expression was performed as previously reported [44]. The harvested cells were washed once with PBS(−) and stained with FITC-conjugated anti-human CD95 (Fas) antibody or FITC-conjugated mouse IgG_1_ isotype control for 30 min at 4 °C in the dark. After staining, the cells were washed and analyzed using flow cytometry (Cytomics FC500; Beckman–Coulter).

### 4.12. qRT-PCR

Total RNA extraction and the synthesis of complementary DNA templates were performed as previously reported [45]. Quantitative RT-PCR was performed using Power SYBR^®^ Green (Applied Biosystems, Inc., Carlsbad, CA, USA) and a StepOnePlus^TM^ system (Applied Biosystems, Inc.) with typical amplification parameters (95 °C for 10 min, followed by 40 cycles of 95 °C for 15 s and 60 °C for 1 min). The relative differences were calculated by the ΔΔ*C*t method. β-actin was used as the housekeeping gene. Primers for caspase-8 and β-actin are shown in Table 1.

### 4.13. Statistical Analysis

Data are presented as mean ± SD. Comparisons between the control and experimental groups were performed using two-sided Student’s *t*-tests or two-sided Mann–Whitney’s *U*-test depending on the data distribution. Differences were considered significant when *p* <0.05. Excel 2016 software (Microsoft, USA) with the add-in software Statcel 4 (The Publisher OMS Ltd., Tokyo, Japan) was used to perform the statistical analyses.

## Figures and Tables

**Table 1:** Primer sequences used for quantitative reverse transcription polymerase chain reaction (qRT-PCR).

Sequence (5′→3′)	
Caspase-8 F	CTTCCTGCCTGCCTGTACC
Caspase-8 R	CGTGCCCAGAAAGTGGAC
β-actin F	TGGCACCCAGCACAATGAA
β-actin R	CTAAGTCATAGTCCGCCTAGAAGCA

## Abbreviations

ATM	Ataxia–telangiectasia mutated
ATR	Ataxia–telangiectasia and Rad-3-related
DMSO	Dimethyl sulfoxide
DNA-PKcs	DNA-dependent protein kinase, catalytic subunit
DSB	Double-strand breaks
ER	Endoplasmic reticulum
HR	Homologous recombination
IAP	Inhibitor of apoptosis protein
MFI	Mean fluorescence intensity
NHEJ	Non-homologous end joining
PARP	Poly ADP ribose polymerase
PI	Propidium iodide
PMA	Phorbol 12-myristate 13-acetate
TRAF2	Tumor necrosis factor receptor-associated factor 2
WB	Wash buffer
XIAP	X-linked inhibitor of apoptosis protein
